# Metabolic correlates of health-related quality of life among overweight and obese adolescents

**DOI:** 10.1186/s12887-018-1044-8

**Published:** 2018-02-03

**Authors:** Chih-Ting Lee, Chung-Ying Lin, Carol Strong, Yu-Fang Lin, Yen-Yin Chou, Meng-Che Tsai

**Affiliations:** 1 Department of Family Medicine, National Cheng Kung University Hospital, College of Medicine, National Cheng Kung University, Tainan, Taiwan; 2 Institute of Physical Education, Health and Leisure Studies, College of Management, National Cheng Kung University, Tainan, Taiwan; 3 Department of Rehabilitation Sciences, Faculty of Health and Social Sciences, Hong Kong Polytechnic University, Kowloon, Hong Kong; 4 Department of Public Health, National Cheng Kung University Hospital, College of Medicine, National Cheng Kung University, Tainan, Taiwan; 5 Department of Pediatrics, National Cheng Kung University Hospital, College of Medicine, National Cheng Kung University, 1 University Road, Tainan, 70101 Taiwan

**Keywords:** Obesity, Adolescent, Health-related quality of life, Cardiometabolic risk

## Abstract

**Background:**

Little is known about the metabolic factors associated with the health-related quality of life (HRQOL) among obese youths. The aim of this study is to assess metabolic correlates of HRQOL in a clinical sample of Taiwanese overweight and obese (OW/OB) adolescents.

**Methods:**

OW/OB adolescents (age 11–19 years) were recruited and compared to their normal-weight counterparts in a tertiary hospital. HRQOL was assessed by the Pediatric Quality of Life Inventory (PedsQL). Student *t* tests and Cohen’s *d* were used to compare the differences in the PedsQL scores between normal-weight and OW/OB participants who were stratified by their cumulative number of cardiometabolic risk factors (CRF). Pearson’s correlation and multivariate linear regression analyses were applied to identify predictors of PedsQL.

**Results:**

OW/OB adolescents (*n* = 60) reported lower PedsQL scores than those of normal-weight peers. The negative effects were even larger in OW/OB participants with more than one CRF. Body mass index z-scores and serum alanine aminotransferase (ALT) levels were negatively correlated with overall and subscales of PedsQL (r = − 0.283 to − 0.431). Multivariate linear models showed ALT to be the most salient factor associated with poor obesity-related HRQOL.

**Conclusion:**

Taiwanese OW/OB adolescents, particularly those having additional CRF, reported worse HRQOL. Impaired liver functions may predispose OW/OB subjects to even worse HRQOL.

## Background

The prevalence of overweight and obese children and adolescents has risen dramatically in the past few decades and has become a health burden worldwide as well as in Taiwan [1, 2]. A recent nationwide survey reported that approximately one third of Taiwanese boys and one quarter of Taiwanese girls aged 6–13 years were overweight or obese [3]. As the trend in the prevalence of obesity in childhood is still on the increase, the physical and psychosocial consequences associated with obesity remains a major concern for health care providers and public health professionals [4].

Childhood and adolescent obesity is associated with a wide range of adverse health outcomes including metabolic syndrome, insulin resistance, diabetes, polycystic ovarian syndrome and cardiovascular diseases [5]. Overweight and obese youths are particularly at risk for an increased incidence of morbidity and mortality in adulthood [6]. The clustering of cardiovascular risks is a hallmark of physical complications related to obesity. Insulin resistance driven by excessive accumulation of adipose tissues, which are relatively inefficient at disposing energy and metabolizing glucose, is considered the main underlying cause of cardiometabolic disorders [7]. While substantial research has been dedicated to investigating the clinical significance of metabolic syndrome in children and adolescents, an increasing prevalence of cardiometabolic risk factors (CRF) is being consistently reported among obese youths, which raises the question of how this trend may affect individuals’ lifelong health status and continues to be a critical topic of research [8].

In addition to these negative effects on physical health, obesity may also affect psychosocial functioning, as well as the overall health-related quality of life (HRQOL), a multidimensional construct for measuring the impact of disease [9]. Several studies have disclosed an inverse association between obesity and pediatric HRQOL [10, 11]. Obese children and adolescents reported lower HRQOL, particularly in the physical and social domains, than their normal-weight peers. This is confirmed in both community and clinical samples [12]. Also, some studies have found that contextual factors such as social and emotional support, family functioning, and peer relationships may influence self-perceived HRQOL [13–15]. Despite a strong link between metabolic syndrome and obesity, few studies have examined the metabolic factors associated with lower HRQOL among obese youths. Conflicting results exist surrounding the correlation between obesity-related comorbidities and HRQOL [14, 16]. Whether and how the results of metabolic surveys, which are routinely conducted on obese youths during clinical examinations, are related to their HRQOL remains unclear. Therefore, the aim of this study is to clarify the relationship between metabolic parameters and HRQOL among Taiwanese obese adolescents. We hypothesized that metabolic derangements related to obesity further impaired the HRQOL for overweight and obese teenagers.

## Methods

### Participants

Adolescents aged 11–19 years with body mass index (BMI) > 85th percentiles for the same-sex-and-age population were consecutively recruited in tertiary hospital-based pediatric endocrine/obesity clinics, where they were referred for the screening of obesity-related endocrine complications. In the meanwhile, patients referred for minor illness or routine health examinations were recruited as normal-weight controls. Explainable genetic or endocrine causes of obesity, such as Down syndrome and hypothyroidism and those with psychiatric illness such as major depression disorder were excluded from the present study. All participants were requested to complete HRQOL and lifestyle questionnaires, and underwent physical examinations including anthropometric measurements. Participants’ BMI were standardized and transformed to z-scores according to age- and sex-specific BMI in the analysis. The participants and their guardians or parents were required to give their informed consents. All the procedures were approved by the Institutional Review Board of National Cheng Kung University Hospital.

### Metabolic investigation

Fasting serum was collected for an analysis of glucose, insulin, triglyceride (TG), total cholesterol, low-density lipoprotein (LDL) cholesterol, high-density lipoprotein (HDL) cholesterol, osteocalcin, C-telopeptides (CTX), and 25-hydroxyvitamin D (25-OHD). The cutoff points of CRFs were defined according to the International Diabetes Federation Consensus [17]. The criteria were blood pressure systolic limits ≥130 or diastolic ≥85 mmHg, fasting glucose ≥100 mg/dL, TG ≥150 mg/dL, and HDL cholesterol ≤40 mg/dL. For females over 16 years of age, the cutoff point for HDL cholesterol was below 50 mg/dL. Insulin sensitivity was represented by homeostasis model assessment-insulin resistance (HOMA-IR) and the quantitative insulin sensitivity check index (QUICKI) [18, 19]. Body composition was measured by dual-energy X-ray absorptiometry.

### Questionnaires on HRQOL and lifestyles

HRQOL was assessed using the Pediatric Quality of Life Inventory (PedsQL), which contains 23 items in four subscales: physical, emotional, social and school. The Taiwan version of PedsQL has been validated and applied in clinical and public health research [20, 21]. Physical activity was assessed using the International Physical Activity Questionnaire (IPAQ).The Taiwan short version has been validated and contains items describing the intensity, frequency and duration of physical activity categorized by a metabolic equivalent (MET), i.e. light (< 3 METs), moderate (3–6 METs)and vigorous (> 6 METs), during a week [22]. Sun exposure and sleep duration were also self-reported by the participants in the questionnaire.

### Statistical analysis

Anthropometric measurements, metabolic parameters and lifestyle characteristics were compared between normal-weight and overweight/obese adolescents using Student *t* tests and χ^2^ tests for continuous and categorical variables, respectively. Levene’s and Kolmogorov-Smirnov tests were used to determine the equality of variance and the normality of distribution, respectively, before conducting independent t tests to compare metabolic parameters and the PedsQL scores between normal-weight and overweight/obese groups. Mann-Whitney U test was applied to the comparison of the variables that were not normally distributed. Moreover, we stratified the overweight/obese group by the cumulative number of CRFs. That is, the normal-weight group was compared with all overweight/obese participants; with overweight/obese participants having more than 1 CRF; and with overweight/obese participants having more than 2 CRFs. In addition to the inferential statistics, we used the Cohen’s effect size (*d*) to portray the magnitude of the discrepancies: the mean difference between groups divided by the standard deviation of pooled PedsQL scores. The *d* < 0.2 indicates trivial; between 0.2 and 0.5 as small; between 0.5 and 0.8 as moderate; > 0.8 indicates large [23]. Partial correlation analyses with adjustment for age and gender were used to identify significant metabolic and lifestyle correlates of PedsQL. Afterward, several multiple linear regression models were constructed to examine the factors associated with PedsQL scores among overweight and obese youths. In model 1, we used the BMI z-score as the independent variable and PedsQL scores as the dependent variable. In model 2, we added metabolic parameters including glucose, lipid, and bone metabolism markers, GPT, insulin sensitivity indices, and total fat percentages as independent variables in the analysis. In model 3, we added lifestyle parameters, such as moderate-vigorous physical activity, sun exposure, and sleep duration as independent variables. A stepwise selection method was used with a significance level of 0.1 set for entry and 0.1 for stay in models 2 and 3. In model 4, we further included and analyzed the interaction effects between BMI and selected variables in model 3 with applying the same stepwise selection criteria. Age and gender were covariates adjusted in all models. All statistical analyses were performed using SPSS 16.0 (SPSS Inc., Chicago, IL, USA).

## Results

A total of 60 overweight and obese adolescents aged 13.3 (±2.3) years, with male predominance (*n* = 40, 66.7%), were recruited with an average BMI of 29.6 (±4.6) kg/m^2^, which corresponded to an average z-score of 3.58 (±1.5). Compared to normal-weight participants, overweight and obese adolescents had higher BMI z-scores, higher fasting insulin, higher HOMA-IR and QUICKI values, higher TG, lower HDL cholesterol, and lower 25-OHD levels (Table 1). Total and trunk fat percentages were much higher in overweight/obese adolescents than those in their normal weight peers (38.6% and 41.2% vs. 15.7% and 14%). Thirteen (21.7%) overweight and obese adolescents had abnormally elevated ALT (> 54 IU/L). Half of them had abdominal echography compatible with fatty liver diseases. The overweight and obese adolescents also spent less time in moderate-to-vigorous physical activity. No difference was found in light exercise, sun exposure and sleep duration between the two groups.

**Table 1:** Metabolic and lifestyle characteristics of participants

	Normal weight (*n* = 10)	Overweight/obese (*n* = 60)	*p*-value
Age, years	14.4 (±2.9)	13.3 (±2.3)	0.178
Male/female, n	7/3	40/20	0.835
BMI, kg/m^2^	17.8 (±2.9)	29.6 (±4.6)	< 0.001
BMI z-score	−0.43 (±0.6)	3.58 (±1.5)	< 0.001
Systolic pressure, mmHg	109.6 (±13.5)	121.8(±14.6)	0.017
Diastolic pressure, mmHg	60.8 (±13.4)	74.8 (±12.7)	0.002
Glucose metabolism markers			
Fasting glucose, mg/dl	86.2 (±4.7)	97.6 (±24.5)	0.148
Insulin, uU/ml	4.2 (±2.6)	17.6 (±15.9)	< 0.001
HOMA-IR	0.89 (±0.58)	4.28 (±4.13)	< 0.001
QUICKI	0.41 (±0.04)	0.32 (±0.03)	< 0.001
ALT, U/l	20.9 (±24.2)	42.6 (±45.8)	0.150
Lipid metabolism markers			
Triglycerides, mg/dl	80.7 (±32.6)	121.8 (±65.8)	0.005
Total cholesterol, mg/dl	167.2 (±38.2)	172.8 (±29.4)	0.592
LDL cholesterol, mg/dl	100.5 (±24.7)	113.9 (±28.4)	0.167
HDL cholesterol, mg/dl	68.0 (±18.4)	49.5 (±10.6)	< 0.001
Bone metabolism			
25-OHD, ng/ml	33.6 (±7.0)	27.9 (±7.3)	0.039
C-terminal telopeptide, pg/ml	1562.6 (±882.3)	1443.3 (±695.5)	0.760
Osteocalcin, ng/ml	99.4 (±45.0)	71.8 (±38.1)	0.068
Body composition			
Trunk fat (%)	14.0 (±7.9)	41.2 (±4.6)	< 0.001
Total fat (%)	15.7 (±7.3)	38.6 (±4.8)	< 0.001
Lifestyle parameters			
Heavy-moderate vigorous exercise, mins/day	138.6 (±95.6)	36.8 (±40.7)	< 0.001
Light vigorous exercise, mins/day	18.0 (±28.5)	17.7 (±41.7)	0.985
Sleep, hours/day	7.6 (±1.3)	8.0 (±1.3)	0.940
Sun exposure, hours/day	2.2 (±1.0)	2.1 (±2.0)	0.404

*BMI* means body mass index, *HOMA-IR* homeostatic model assessment for insulin resistance, *QUICKI* quantitative insulin-sensitivity check index, *ALT* alanine aminotransferase, *HDL* high-density lipoprotein, *LDL* low-density lipoprotein 25-OHD, 25-hydroxyvitamin D

As compared to normal-weight controls, overweight and obese adolescents manifested poorer PedsQL scores in overall, physical, and psychosocial domains (Table 2). The effect sizes were moderate with a Cohen’s *d* ranging from 0.5 to 0.8. A total of 70% of the overweight and obese adolescents developed at least one additional CRF, and the one-third met the criteria for metabolic syndrome, which is defined by more than 2 CRFs associated with obesity. Further analyses stratifying the participants by the cumulative number of CRFs showed that those who have CRFs manifested poorer PedsQL scores, except for those in the emotional subdomains of the PedsQL.

**Table 2:** Comparison of PedsQL domain scores among normal weight and overweight/obese adolescents stratified by the cumulative number of cardiometabolic risk factors

PedsQL	Normal weight	Overweight/obese^a^					
	(*n* = 10)	overall (n = 60)		≥ 1 CRF (*n* = 42)		≥ 2 CRF (*n* = 20)	
	Mean (±S.D.)	Mean (±S.D.)	Effect size^b^ (*p*-value)	Mean (±S.D.)	Effect size^b^ (*p*-value)	Mean (±S.D.)	Effect size^b^ (*p*-value)
Total score	90.7 (±10.2)	79.2 (±17.3)	0.67* (0.009)	77.9 (±18.7)	0.75* (0.007)	77.4 (±19.3)	0.74* (0.021)
Physical	91.9 (±11.2)	81.8 (±15.1)	0.67* (0.048)	80.4 (±16.2)	0.74* (0.041)	81.8 (±16.8)	0.64 (0.101)
Psychosocial	90.0 (±10.7)	77.9 (±19.6)	0.64* (0.009)	76.5 (±21.1)	0.75* (0.008)	75.1 (±21.9)	0.75* (0.018)
Emotional	86.0 (±15.6)	76.2 (±21.7)	0.47 (0.174)	74.4 (±23.6)	0.53 (0.146)	71.0 (±25.8)	0.63 (0.103)
Social^c^	97.0 (±7.9)	84.6 (±22.9)	0.57* (0.027)	84.4 (±21.7)	0.66* (0.026)	84.5 (±21.0)	0.67 (0.055)
School	87.0 (±15.7)	72.9 (±23.8)	0.61 (0.076)	70.8 (±26.0)	0.67* (0.019)	69.8 (±25.0)	0.73* (0.029)

*PedsQL* represents the Pediatric Quality of Life Inventory 4.0, *S.D.* standard deviation

^a^18 overweight/obese children did not have cardiometabolic risk factor

^b^Effect size using Cohen’s *d*: difference of two samples divided by pooled standard deviation. Effect size between 0.5 and 0.8 indicates moderate effect

^c^Mann-Whitney U test was applied because the PedsQL scores in the social domain were not normally distributed in the normal weight group

*p-value < 0.05

In the partial correlation matrix (Table 3), total PedsQL scores were negatively correlated with BMI z-scores (*r* = − 0.287, *p* = 0.017) and ALT levels (r = − 0.363, *p* = 0.002). Scores in the physical domain were negatively correlated with BMI z-scores(r = − 0.338, *p* = 0.005), ALT levels (r = − 0.431, *p* < 0.001), and moderate-to-vigorous physical activity (r = − 0.253, *p* = 0.038). ALT levels were also negatively correlated with PedsQL in all psychosocial domains except for the school domain, while BMI z-scores were negatively correlated with the school domain.

**Table 3:** Correlation matrix of body mass index, metabolic and lifestyle factors, and PedsQL^a^

	Total score	Physical	Psychosocial	Emotional	Social	School
BMI z-score	−.287*	−.338**	−.250*	−.173	−.206	−.266*
Systolic blood pressure	−.144	−.132	−.141	−.135	−.132	−.102
Diastolic blood pressure	−.073	−.042	−.082	−.060	−.114	−.042
Fasting glucose	.036	.049	.029	.011	.058	.006
Insulin	−.143	−.198	−.112	−.111	−.095	−.087
HOMA-IR	−.134	−.184	−.106	−.107	−.081	−.087
QUICKI	.098	.159	.067	.061	.041	.071
ALT	−.363**	−.431***	−.314**	−.315**	−.283*	−.223
Triglycerides	−.018	.005	−.026	−.023	.028	−.069
Total cholesterol	−.093	−.150	−.065	.009	−.159	−.020
LDL cholesterol	−.156	−.197	−.130	−.055	−.183	−.100
HDL cholesterol	.064	−.002	.087	.070	−.045	.191
25-OHD	.043	.010	.056	.015	.047	.086
C-terminal telopeptide	−.173	−.121	−.185	−.272	−.008	−.199
Osteocalcin	−.016	.165	−.097	−.100	−.033	−.095
%Trunk fat	−.174	−.183	−.160	−.090	−.117	−.201
%Total fat	−.151	−.156	−.140	−.072	−.091	−.193
Moderate-to-vigorous PA	.141	.253*	.087	−.022	.075	.163
Light PA	−.060	.029	−.093	−.232	−.034	.012
Sleep duration	.034	.047	.027	.037	.107	−.065
Sun exposure	−.096	−.137	−.074	−.051	.022	−.155

^a^The analysis was adjusted for age and gender

*p < 0.05; **p < 0.01;***p < 0.001

*PedsQL* represents the Pediatric Quality of Life Inventory 4.0, *BMI* body mass index, *HOMA-IR* homeostatic model assessment for insulin resistance, *QUICKI* quantitative insulin-sensitivity check index, *ALT* alanine aminotransferase, *HDL* high-density lipoprotein, *LDL* low-density lipoprotein, *25-OHD* 25-hydroxyvitamin D, *PA* physical activity

Multiple regression analyses showed predictive estimates of metabolic and lifestyle parameters on the PedsQL scores (Table 4). After controlling for age and gender, the BMI z-score was a significant predictor in the total, physical domain, and psychosocial domain scores of PedsQL. After adding metabolic and lifestyle parameters in the analysis of model 3, a significant effect of ALT levels on PedsQL scores was found, explaining 19% of the variance in the physical domain in particular. The elevation of ALT levels contributed to poorer PedsQL scores in the overall, physical and psychosocial domains, except for the school domain. An analysis of the interaction between BMI and ALT levels in model 4 shows that there is a significant interaction between these 2 factors.

**Table 4:** Linear regression estimates for PedsQL scores according to multivariate models

	Total score		Physical		Psychosocial		Emotional		Social		School	
	β	*t*	β	*t*	β	*t*	β	*t*	β	*t*	β	*t*
Model 1	R^2^ = 0.106		R^2^ = 0.156		R^2^ = 0.077		R^2^ = 0.061		R^2^ = 0.085		R^2^ = 0.071	
BMI z-score	−.28*	−2.43	−.33**	−2.89	−.25*	−2.09	−.17	−1.44	−.20	−1.70	−.26*	− 2.23
Model 2	R^2^ = 0.197		R^2^ = 0.315		R^2^ = 0.146		R^2^ = 0.137		R^2^ = 0.142		R^2^ = 0.071	
BMI z-score	−.21	−1.82	−.33**	−2.80	−.18	− 1.56	−.10	− 0.88	−.14	− 1.20	−.26*	− 2.23
ALT	−.31**	− 2.74	−.38**	−3.54	−.27*	− 2.31	−.29*	− 2.41	−.25*	−2.10		
HDL cholesterol	n.s.		−.21	−1.76	n.s.		n.s.		n.s.		n.s.	
Model 3	R^2^ = 0.197		R^2^ = 0.346		R^2^ = 0.146		R^2^ = 0.137		R^2^ = 0.142		R^2^ = 0.071	
BMI z-score	−.21	−1.82	−.26*	−2.12	−.18	−1.56	−.11	−0.95	−.14	− 1.20	−.26*	− 2.23
ALT	−.31**	−2.74	−.39***	−3.67	−.27*	− 2.31	−.29*	− 2.41	−.25*	−2.10	n.s.	
HDL cholesterol	n.s.		−.21	−1.79	n.s.		n.s.		n.s.		n.s.	
Moderate-to-vigorous PA	n.s.		.20	1.76	n.s.		n.s.		n.s.		n.s.	
Model 4	R^2^ = .260		R^2^ = .383		R^2^ = .208		R^2^ = .184		R^2^ = .201		R^2^ = 0.071	
BMI z-score	−.40**	−2.84	−55***	−3.78	−.37**	−2.59	−.27	−1.83	−.32*	− 2.20	−.26*	−2.23
ALT	−.77**	−3.23	−.83***	−3.72	−.74**	−2.99	−.70*	−2.80	−.68**	− 2.73	n.s.	
HDL cholesterol	n.s.		−.26*	−2.29	n.s.		n.s.		n.s.		n.s.	
Moderate-to-vigorous PA	n.s.		n.s.		n.s.		n.s.		n.s.		n.s.	
BMI z-score x ALT	.59*	2.19	.58*	2.32	.60*	2.15	.53	1.87	.57	1.99	n.s.	
BMI z-score x HDL cholesterol	n.s.		n.s.		n.s.		n.s.		n.s.		n.s.	
BMI z-score x Moderate-to-vigorous PA	n.s.		n.s.		n.s.		n.s.		n.s.		n.s.	

*p < 0.05; **p < 0.01;***p < 0.001

Model 1 included only BMI z-score and age in the analysis. Model 2 added metabolic factors with stepwise methods in the analysis. Model 3 added metabolic and lifestyle factors with stepwise methods in the analysis

Model 4 further included three interaction terms between BMI z-score and ALT, HDL cholesterol and moderate-to-vigorous PA with stepwise methods in the analysis

*PedsQL* represents the Pediatric Quality of Life Inventory 4.0, *BMI* body mass index, *ALT* alanine aminotransferase, *HDL* high-density lipoprotein, *PA* physical activity, *n.s.*, not significant

## Discussion

Our results demonstrated that medically referred overweight and obese adolescent patients presented a cluster of metabolic abnormalities and also reported lower HRQOL, particularly among those having additional cardiometabolic risk. Although substantial research exists showing the negative impacts of obesity on HRQOL [10, 12, 13], our study extended the current knowledge to include medically referred young patients in a local Taiwanese context and added clinical implications for the health care of pediatric obesity.

Contrary to previous research [14, 16], our results showed that the cumulative number of cardiometabolic risk factors was associated with greater impairment in HRQOL. In an earlier study conducted in the United States, approximately two-thirds of participants had obesity-related comorbidity, but the existence of comorbidity, such as hypertension and dyslipidemia, did not account for the way obesity affects HRQOL as measured by the PedsQL [14]. However, the parent-proxy PedsQL score was inversely associated with the amount of comorbidity. This association remained significant after multivariate adjustments, which is quite comparable to our results. Although they only saw a weak correlation between adolescent and parent-proxy assessments of HRQOL, the parents’ reports might be able to reflect additional perspectives on the health impact of obesity [14, 21, 24]. In another Dutch study of a cohort of exclusively severely obese cases, nearly 80% of participants had more than one CRF in addition to obesity; yet they did not report lower HRQOL measured using EuroQol utility scores [16]. The differences in research findings could be explained by variations in the measures and analytic methods used in the studies. EuroQol utility scores principally measure utility decrements caused by the diseases and health problems under investigation [25]. In such cases the cardiometabolic risks or comorbidities in obese youth are symptomatically mild and thus the impacts may not be fully represented in the utility-based HRQOL scale. The PedsQL that we applied in this study has been psychometrically validated and is sensitive to evaluate both physical and psychosocial HRQOL. Our results may suggest that obese subjects who have additional cardiometabolic risks are more prone to adverse effects of obesity on their HRQOL, despite their general lack in daily functional impairments. From a clinical perspective, health care providers should be aware of the obesity-related cardiometabolic comorbidities and their reciprocal influence on HRQOL, given the high prevalence of these risk factors in obese youths. Continual monitoring of these parameters, as well as counseling on the psychosocial impacts, should be provided in the medical services dedicated to adolescent obesity.

Further dissecting the relationship between metabolic factors and HRQOL, we identified greater negative impacts on many aspects of HRQOL in obese youths with impaired liver functions. In this cohort, greater BMI was associated with worse HRQOL; yet the significance of the association was attenuated, except in the physical domain, when adjusting for metabolic and lifestyle factors. In contrast, ALT levels became the most salient predictor of poor HRQOL in all except the school domain. In addition, abdominal echography found that non-alcoholic fatty liver disease (NAFLD) was a complication commonly encountered in obese patients with elevated liver enzymes. These findings were consistent with previous research on adult hepatitis and HRQOL showing that impairment in HRQOL was noted in patients with NAFLD but less pronounced than that in patients with viral hepatitis [26]. Weight reduction could improve HRQOL among NFLAD patients despite a lack in biochemical amelioration [27]. We hypothesized that the cause of poor HRQOL in association with elevated liver functions may be related to fatigue, which is a common complaint in hepatitis patients [28]. Moreover, evidence in the existing literature also argues that non-specific psychological symptoms such as depressive moods, notably found in viral hepatitis, may partly explain impairment in HRQOL [29]. Taken together, the results of this study highlighted the complexity of the adverse impacts caused by obesity on children’s and adolescents’ HRQOL. Beyond the psychosocial link, disturbances in the neurotransmission system potentially account for the emotional or cognitive maladjustment experienced by obese youths [30]. As previous research suggested that weight loss might result in improvements in HRQOL [31], we are keen to see whether there is a contributing role of metabolic recovery in changes of HRQOL after weight reduction in these patients. Further research is required to elucidate the biological mechanism and its clinical implications.

This study has some limitations. First, our cohort of participants was exclusively recruited from medically referred patients. They presumably represented severe cases that may have higher percentages of obesity-related metabolic complications. Selection bias may exist when recruitment only takes place in a tertiary hospital, as emerging research has suggested that treatment-seeking pediatric populations may have poorer HRQOL than that obtained from community samples [32]. Although the metabolic risks are prevalent among community-based obese youths, how these factors interact with obesity and thus impact on HRQOL in a community setting remains unaddressed. The generalization of these results derived from a small clinical sample may require further research. Second, the genetic predisposition to metabolic syndrome beyond obesity was not evaluated in our analysis. For example, parental health status and the family history of metabolic diseases were not included. Early onset of familial metabolic diseases may bias the correlation between metabolic factors and HRQOL, although this is usually rare. Obtaining parental biochemical data may help elucidate this genetic concern. Third, only patient reports of HRQOL were used and thus could be biased if the participants failed to recognize or exaggerated their health problems. The inclusion of both parent-proxy and children-reported HRQOL measurements is suggested to gain a complete picture of functioning [33]. The use of generic HRQOL tool can be less sensitive to detecting decrements in obesity-specific HRQOL. Replication studies with the application of HRQOL specifically developed for childhood and adolescence obesity, such as Sizing Me Up and Sizing Them UP, may be needed to consolidate our findings [34, 35]. Moreover, age and gender have been shown to be important determinants of HRQOL in children and adolescents [33]. Because of the small participation size in this study, we could not stratify our cohort of participants to see the differential effects inherent to age and gender. Other potential confounding factors which may affect HRQOL, such as emotional/social support, family functioning, self-perception of obesity, teasing and bullying experience and interpersonal relationships were not evaluated in the current analysis. Further studies with a larger number of participants may be needed to explore the stratification effect of potential confounders.

## Conclusions

Overweight and obese adolescents presented several metabolic abnormalities in our pediatric clinics. Having additional cardiometabolic risk factors appeared to contribute to worse outcomes in HRQOL. Also, elevated liver functions may partially explain the link between observed obesity and impairment in HRQOL. The results may suggest a biological mechanism underlying the cause of psychosocial maladjustments related to obesity. Clinicians and health care providers should therefore be aware of adverse psychosocial impacts associated with cardiometabolic derangements in obese patients.

## Abbreviations

25-OHD: 25-hydroxyvitamin D
ALT: Alanine aminotransferase
BMI: Body mass index
CRF: Cardiometabolic risk factors
CTX: C-telopeptides
HDL: High-density lipoprotein
HOMA-IR: Homeostasis model assessment-insulin resistance
HRQOL: Health-related quality of life
IPAQ: International physical activity questionnaire
LDL: Low-density lipoprotein
MET: Metabolic equivalent
OB: Obese
OW: Overweight
PedsQL: Pediatric quality of life inventory
QUICKI: Quantitative insulin sensitivity check index
TG: Triglyceride
